# Sulfur nutrition and its role in plant growth and development

**DOI:** 10.1080/15592324.2022.2030082

**Published:** 2022-02-07

**Authors:** Om Prakash Narayan, Paras Kumar, Bindu Yadav, Meenakshi Dua, Atul Kumar Johri

**Affiliations:** a School of Life Sciences, Jawaharlal Nehru University, New Delhi, India; b School of Environmental Sciences, Jawaharlal Nehru University, New Delhi, India

**Keywords:** Sulfur, Sulfur transport, Sulfur metabolism, Sulfur assimilation, Plant growth

## Abstract

Sulfur is one of the essential nutrients that is required for the adequate growth and development of plants. Sulfur is a structural component of protein disulfide bonds, amino acids, vitamins, and cofactors. Most of the sulfur in soil is present in organic matter and hence not accessible to the plants. Anionic form of sulfur (SO_4_^2−^) is the primary source of sulfur for plants that are generally present in minimal amounts in the soil. It is water-soluble, so readily leaches out of the soil. Sulfur and sulfur-containing compounds act as signaling molecules in stress management as well as normal metabolic processes. They also take part in crosstalk of complex signaling network as a mediator molecule. Plants uptake sulfate directly from the soil by using their dedicated sulfate transporters. In addition, plants also use the sulfur transporter of a symbiotically associated organism like bacteria and fungi to uptake sulfur from the soil especially under sulfur depleted conditions. So, sulfur is a very important component of plant metabolism and its analysis with different dimensions is highly required to improve the overall well-being of plants, and dependent animals as well as human beings. The deficiency of sulfur leads to stunted growth of plants and ultimately loss of yield. In this review, we have focused on sulfur nutrition, uptake, transport, and inter-organismic transfer to host plants. Given the strong potential for agricultural use of sulfur sources and their applications, we cover what is known about sulfur impact on the plant health. We identify opportunities to expand our understanding of how the application of soil microbes like AMF or other root endophytic fungi affects plant sulfur uptake and in turn plant growth and development.

## Introduction

**Figure 1.**
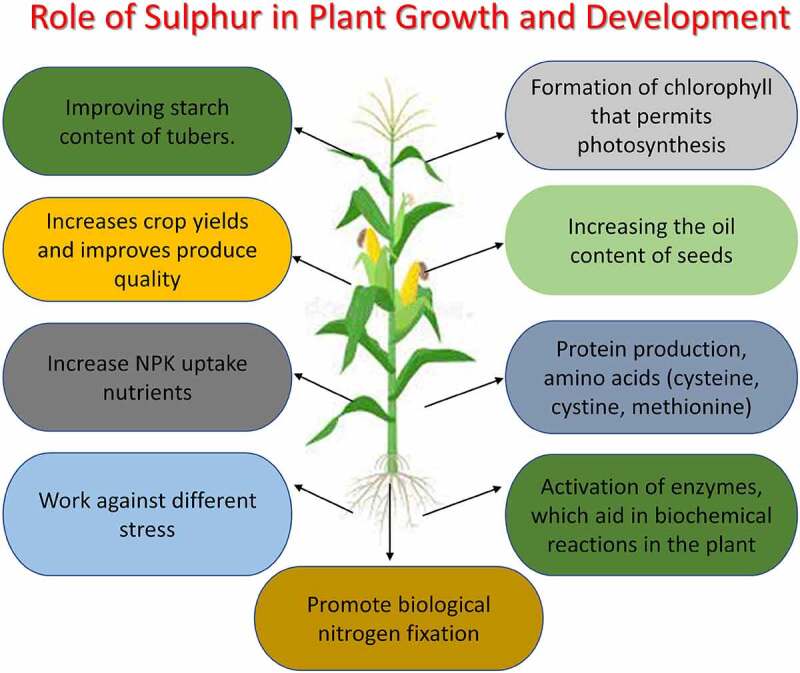
Schematic representation of the role of sulfur in plant growth and development.

Sulfur is one of the essential elements required by all living organisms, including plants. Sulfur is a constituent of the proteinaceous amino acids such as methionine and cysteine, glutathione, vitamins (biotin and thiamine), phytochelatins, chlorophyll, coenzyme A, and *S*-adenosyl-methionine.^1–3^ Sulfur is also involved in disulfide bond formation in proteins and enzymes’ regulation, particularly in redox control. It offers protection from oxidative damage through glutathione and its derivatives.^4^^,^^5^ Sulfur is also a component of several secondary metabolites (SMs) of plants and is required for the plant’s physiological functions, growth, and development. The sulfur demand in plants is dependent on the types of species and stages of development. For instance, during seed development and vegetative growth, a higher amount of sulfur is required.^6^ Sulfur-containing compounds such as Fe–S clusters-containing proteins are required in multiple biological processes, such as photosynthesis, energy generation, photoprotection, and metabolic reactions.^7,8,9^ The primary and dominant sulfur source is inorganic sulfate (SO_4_^2−^) for the plants.^1^^,^^10^ Multiple transporters are involved in SO_4_^2−^ uptake and its transportation from source to sink. Chloroplasts of young leaves are the prominent organelle where assimilation of SO_4_^2−^ to cysteine occurs; however, synthesis of methionine and cysteine can also happen in seeds and roots.^4^^,^^11^ Moreover, in plants, sulfur, and sulfur-containing compounds are directly or indirectly take part in biotic and abiotic stress management, metabolism, and signaling. The overall role of sulfur in plant growth and development is summarized in Figure 1.

## Sources of sulfur in soil

There are various sources of sulfur found in the soil. Organic matter contains around 95% of the total sulfur content of the soil (https://blog-crop-news.extension.umn.edu/2018/01/evaluating-need-for-sulfur-in-high.html).^12^ Breakdown or decomposition of organic matter results in mineralization of organic sulfur into the SO_4_^2−^, which will be available to plants.^13^ Apart from the organic matter, various minerals inside the soil also consist of a different sulfur forms. Hence, breaking down or weathering these minerals results in transforming a part of sulfur into sulfate.^14^ In the atmosphere, a higher concentration of SO_2_ is observed around the industrial area. Fuel-burning is also a source of sulfur. It releases sulfur in the form of sulfur dioxide (SO_2_).^15^ This SO_2_ is dissolved in rainwater and finally reaches the soil. Pesticides contribute comparatively small amounts of sulfur to the soil.^16^^,^^17^ However, some pesticides contain sulfur, and the use of pesticides adds sulfur to the soil.^17^ Chemical fertilizers contain a considerable amount of sulfur along with nitrogen, potassium, and phosphorus.

## Sulfur deficiency in plants

Sulfur deficiency results in poor quality and yield of crops.^18^^,^^19^ Mild sulfur deficiency may have a negligible effect on yield but have a significant impact on quality. Thus, poor or low sulfur storage proteins are synthesized in sulfur limiting soil, such as omega-gliadin and high molecular weight subunits of glutenin at the expense of sulfur-rich proteins in wheat.^20^^,^^21^ It has been reported that sulfate deficiency leads to decreased synthesis of Rubisco (ribulose-1,5-biphosphate carboxylase/oxygenase) enzyme that affects the assimilation rates of CO_2_ which eventually results in retarded synthesis of carbohydrates this resulted in the chlorosis of young leaves.^20–22,23^ Several studies suggest that sulfur deficiency affects biomass, overall morphology, yield, and nutritional value of the plants. For instance, in *Eruca sativa* L sulfur deficiency leads to altered biomass production and chlorophyll synthesis.^25^ Moreover, the impacts of sulfur supplementation on grain yield and protein yield of agronomically important traits in wheat were observed in a study. The average protein yield of different cultivars was increased from 0.018 to 0.024 kg m^−2^ and the average grain yield of different cultivars was increased from 0.20 to 0.29 kg m^−2^.^21^ In another study, it has been observed that impact of sulfur supplementation improve grain yield and protein yield in agronomically important plants like wheat and oilseed rape.^26^ Additionally, sulfur deficiency leads to decreased root hydraulic conductivity as a response probably implicated with signaling nutrient starvation from root to shoot.^24^^,^^27^ Moreover, sulfur deficiency results in the reduction of the internal sulfur pool and an increase in the soluble nitrogen pool together with amide and nitrate as a consequence of the ratio of nitrogen and sulfur imbalance.^27^^,^^28^ Sulfur deficiency symptoms in economically important plants are summarized in Table 1.

**Table 1.** Sulfur deficiency symptoms in economically important plants.

Plants	Symptoms
**Wheat**	Yellowing of the plant, more prominent between the veins.
**Rice**	Yellowish leaf sheath and leaf blade. Reduced plant height and number of tillers. Fewer panicles, shorter and fewer grains.
**Maize**	The initial stage, yellowing between the veins in younger leaves. Later, reddening at the base of the stem and along the leaf margins.
**Chickpea**	Plants appear erect, premature drying, and withering of young leaves.
**Sunflower**	Leaves and flowers become pale. Plants are smaller with shorter internodes. Reduced number and size of leaves.
**Tomato**	Small plant height and lighter green. Yellowing in various plant parts. In the severe deficiency, petioles and stems show a clear reddening.
**Groundnut**	Small Plant height. A “V” shaped petiole appearance. New leaves, the area around the main vein may be pale. Seed maturity delayed.
**Sugarcane**	Younger leaves become yellowish-green colors. Older leaves show a faint purplish tinge. Stems are thinner and taper toward the tip.
**Tea**	Sulfur deficient bushes turn yellow, reduce in leaf size, short internodes, the entire plant appears shrunken. Leaves curl up and their edges and tips turn brown.
**Pea**	Chlorosis in young leaves. Flowering and yield are reduced.
**Tobacco**	Young leaves are uniformly pale-yellow green. Leaves are smaller and internodes are shorter.
**Banana**	Young leaves show chlorosis. Severe sulfur deficient conditions lead to chlorosis in between the veins. Retracted growth and small fruits are produced.
**Green** **gram**	Stunted plants growth reduced branching and flowering, and pods have shrunken seeds.
**Cotton**	Persistent yellowing of new leaves and reddening of the petiole.
**Potato**	Evident inward curling of youngest leaves, substantial yellowing of the stems, overall yellowing of the plants
**Coffee**	Young leaves show yellow color, mature leaves show chlorosis of mature, small leaves size. Interveinal tissue looks like a mottled appearance.
**Rubber**	The entire leaf surface turns yellowish-green color, reduced in size, with typical brown necrotic spots at the tips of the leaves.

Information in this table was adopted form The Sulfur Institute (TSI), Washington DC, USA (Sulfur Deficiency Sources and Symptoms – The Sulfur Institute).

## Factors affecting sulfur deficiency in plants

Sulfur deficiency is more prevalent in recent years because of the reduction in atmospheric inputs. Reduced industrial sulfur emissions because of pollution control regulation resulted in the reduced disposition of sulfur into the soil from the atmosphere (https://www.dekalbasgrowdeltapine.com/en-us/agronomy/the-importance-of-sulfur-for-corn-and-soybeans.html).^15^^,^^25^^,^^29^ In addition, extensive use of high purity and sulfur-free or low percentage sulfur-containing fertilizers/pesticides and intensive production of higher-yielding crops may also contribute to more sulfur deficiency in the soil.^1^^,^^16^^,^^17^^,^^30^^,^^31^ It is reported that between 1990 and 2011 the atmospheric concentration of SO_2_ has been decreased by 20 teragram.^32^ It has been reported that soil factors also affect sulfur deficiency. Organic sulfur is the primary source of sulfur utilized by plants. Therefore, the soil’s organic content is crucial, and if it is low, it will lead to a sulfur deficiency in plants. Organic sulfur becomes available to the plants through mineralization that is carried out by microorganisms.^33^^,^^34^ This microbial activity is dependent on the temperature of the soil as well as the moisture content. Microbial activity is reduced by cold and excessively wet or dry conditions, thereby decreasing sulfur availability from soil organic matter to the plants.^30^^,^^33^ The lack of sulfur can be highly variable at the field level because soil sulfur availability differs significantly from soil organic matter and texture. Sulfur deficiency is frequently seen in sandy soil, lower organic matter, and higher elevation areas of a field. However, high organic matter, lower-lying, and heavier textured areas typically have sufficient sulfur.^35^

## Application of fertilizers to overcome the sulfur deficiency

There are several ways to overcome the sulfur deficiency. Chemical fertilizer, Farmyard Manure (FYM), compost, or organic matter can be used to overcome the sulfur deficiency. There are more than 20 different sulfur-containing fertilizers are available commercially which is immediately available for plant uptake.^36^ A list of percent sulfur content in different chemical fertilizers is given in Table 2.

**Table 2.** Sulfur-containing fertilizers and their approximate composition.

Fertilizer	Percentage of Sulfur
Ammonium sulfate	24
Ammonium thiosulfate	26
Elemental sulfur	>90
Gypsum (calcium sulfate)	19
Potassium magnesium sulfate	23
Potassium sulfate	18

This data was adopted from Purdue University Department of Agronomy, as soil fertility update.^37^

Ammonium thiosulfate is used with either solution of urea-ammonium nitrate or by the mixture of ammonium sulfate and urea. Sulfate of potash magnesia or potassium sulfate can be added to muriate of potash to provide sulfur and potassium. But sulfur fertilizers should be applied to crop that requires sulfur to avoid the chances of leaching from the root zone. Since these fertilizers are used before planting, sulfate can be leached from sandy soil before crop requirement.^30^ A recent study on wheat showed that the use of sulfur-containing fertilizers accelerated their germination as well as an improved immune response against pathogens.^38^

## Role of Arbuscular Mycorrhiza Fungi (AMF) in sulfur supply

AMF are soil-borne fungi that colonize with plant roots. AMF form vesicles, arbuscules, and hyphae in roots, and also, they extend their hyphae in the rhizosphere. AMF works as a bio-fertilizers that improves plant growth by improving water and mineral nutrient uptake from soil rhizosphere.^39^ Several studies on AMF have emphasized their numerous advantages on crop productivity and soil health. Thus, it is believed that AMF could be considered as a substitute for inorganic chemical fertilizers.^40^

Fertilizers can be an option for sulfur supplements during deficiencies. However, the timing and type of sulfur application influence the presence of sulfur in the soil and the availability of the plant. AMF shows a symbiotic association with gymnosperm, angiosperm, fern, and lycopod.^40^^,^^41^ Intra-radical hyphae (IH) of AMF offer fungal extension inside the host plants’ cortical region. In contrast, extra-radicular hyphae (ERH) consist of three primary functions: infection of host plants, nutrient acquisition, and production of fertile spores.^40–43^ Many reports show that AMF colonization with plants increases the sulfur content of plants by increasing its uptake from the soil. During sulfur limitation, plants absorb SO_4_^2−^ very rapidly, which leads to the formation of the SO_4_^2−^ depletion zone.^46^ In such conditions, the AMF ERH can enlarge and extend across the region of SO_4_^2−^ depletion and could be a contributing factor mainly in the provision of sulfur under sulfur limitation conditions.^47^ Moreover, recent findings have shown that the colonization by AMF also influences plant sulfate transporters’ expression, thereby increasing the host plant’s sulfur content.^48^ AMF hyphae provide a large surface area compared to the plant roots, which act as an important site for microbial interactions that play an essential role in nutrient cycling.^49^ It has shown that an AMF like root endophytic fungus *Serendipita indica* helped maize plants to uptake sulfate, particularly under sulfur-deprived conditions.^44^

For soil fertility and plant viability, various microbial communities are required.^40^^,^^50^ It has been shown that AMF hyphae contain higher sulfonate desulfurizing bacterial communities than bulk soil.^51^ In another study, AMF inoculated with *Lolium perenne* showed a significant increase in the colonization of root and cultivable sulfonate mobilizing bacterial colonies, helping sulfur supply to the plant.^52^ Similarly, the addition of 2-(N-morpholine)-ethane sulfonic acid (MES) to soil has been found to stimulate not only sulfonate mobilizing bacteria but also the metabolites of this bacteria which are considered to have important role in the growth improvement of ERH of *Glomus intraradices*.^53^ This is essential for increasing the sulfur uptake as enriched hyphal growth arises from sulfonate mobilizing bacterial metabolites stimulates the propagation of this bacterial community in a positive feedback loop. Therefore AMF has a crucial function in plant sulfur metabolism in up-regulating plant sulfate transporters via interaction with organo-sulfur mobilizing microbes. Like the rhizosphere, the AMF hyposphere functions as a region for elevated bacterial activity and its abundance.^40^^,^^52–54^ It is not recognized whether the associated microbes transfer sulfur to the host plant and its symbiont fungi. Plant roots, mycorrhizal hyphae, and several other microbes release extracellular sulfatases into the soil rhizosphere. Although there is no direct evidence of the transfer of sulfur to the plant host through the ERH of AMF; the possibility of release of sulfur indirectly from sulfonate desulfurizing bacteria still exists and can be increased in number by staying away from its predators such as protozoa and nematodes.^55–57 58 59^

Altogether, there is a great need to understand the pathways required to mobilize sulfonates and sulfate-esters that are dominantly present as a chief source of sulfur in the soil. The humic material can be depolymerized by the saprotrophic fungi resulting in the release of sulfate-esters to fungi and bacteria, and sulfonates to the special type of bacteria consist of a monooxygenase enzyme complex. Since desulfurizing microbial populations enriches the rhizosphere and hyphosphere, and hence released SO_4_^2−^ gets assimilated very rapidly, resulting in a sulfur diminished region in the rhizosphere. The percentage of the root colonization and the extent of the sulfonate mobilizing bacterial community has been known to increase due to the inoculation with AMF. Therefore, crop yield can be sustainably improved by inoculation practices in those areas where sulfur is becoming a limiting factor for plant growth.

## Uptake, transport, and assimilation of sulfate

Various membrane transporters help out plants with sulfate uptake from soil and its distribution inside plant cells. Transport across the plasma membrane is eased by a proton gradient maintained by a proton ATPase. The Symport mechanism involves the entry of H^+^ along with sulfate. Sulfate transportation across the tonoplast membrane is managed by the electrical gradient in-between the cytoplasm and vacuole sap. The inner membrane of the chloroplast contains an H^+^/ SO_4_^2−^, which may mediate SO_4_^2−^ transport into chloroplasts.^1^^,^^60^

It has been reported that sulfur status of plants regulates the expression of most of the sulfate transporters. According to the functional, cellular, and subcellular expression, the sulfate transporter gene family consists of five different groups.^1^^,^^11^^,^^61^^,^^62^ *Group 1*: These are high-affinity sulfate transporters, and hence, these sulfate transporters are implicated with the uptake of sulfate by the roots; *Group 2*: These are low-affinity vascular sulfate transporters; *Group 3*: Transporters of this group also known as ‘leaf group’; *Group 4*: These transporters are associated with the sulfate uptake into the plastids before its reduction; *Group 5*: Role of sulfate transporters of this group is still not much studied.

In plants, cystine is the precursor and the sulfur donor for the synthesis of various organic sulfur compounds; hence, the majority of sulfate taken up by the roots is further reduced to sulfide to convert it into cysteine.^4^^,^^61^ The reduction process mainly takes place in the chloroplasts, and this process involves three main steps. First, activation of sulfate to adenosine 5’-phosphosulfate (APS) in the presence of an enzyme ATP sulfurylase. Second, activated APS is reduced to sulfite using APS reductase as an enzyme and glutathione as a reductant.^11^^,^^63^ Third, reduction of sulfite using sulfite reductase enzyme and reduced ferredoxin as a reductant. Sulfide afterward incorporates into cysteine in a reaction catalyzed by O-acetyl serine (thiol) lyase, with O-acetyl serine as the substrate. O-acetyl serine formation is catalyzed by serine acetyltransferase and together with O-acetyl serine(thiol)lyase, associated with cysteine synthase. It has been shown that cysteine synthesis is an important reaction in the direct coupling between sulfur and nitrogen metabolism in plants.^21^^,^^64^ The sulfate reduction pathway is regulated by adenosine phosphosulfate reductase since this enzyme activity is lowest among the enzymes involved in the assimilatory sulfate reduction pathway.^11^^,^^61^ Furthermore, allosteric inhibition and metabolite activation or repressions of the genes encoding the APS reductase are involved in the expression regulation of this enzyme. Thus, both the activity and the expression of APS reductase alter quickly in response to either sulfur starvation or the presence of reduced sulfur compounds. Cysteine, glutathione, Sulfide, or O-acetyl serine are probably APS reductase regulators.^1^^,^^65^

**Figure 2.**
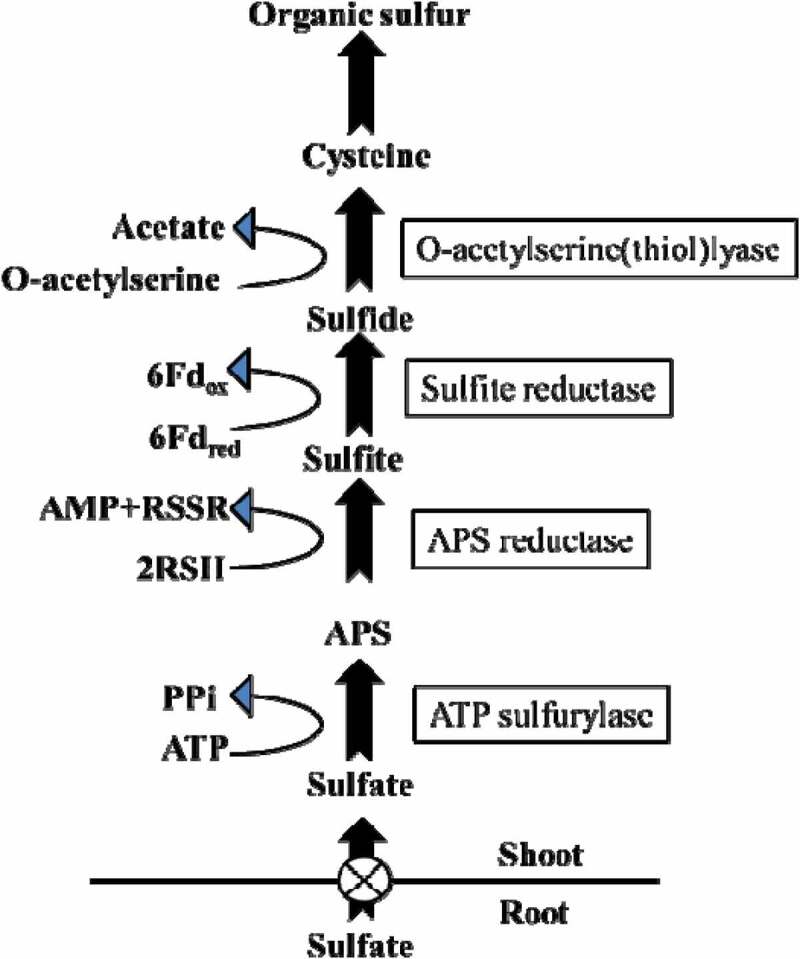
Sulfate reduction and assimilation pathway in plants. (adopted from the Ph.D. thesis, Om Prakash Narayan, school of life sciences, Jawaharlal Nehru University (2018).

Though stress decreases the plant’s growth to a very low level as sulfur is limited, a detectable sulfur level in sulfur deficit plants is reported.^24^^,^^66^ Methionine and other major sulfur-containing amino acids use cysteine as the reduced sulfur donor for their synthesis via the so-called trans-sulfurylation pathway.^60^^,^^67^ Glutathione, phytochelatins, and secondary sulfur compounds also use cysteine as the direct precursor for their synthesis.^61^^,^^68^ The sulfide residue of cysteines in proteins plays a significant role in the binding of enzymes with the substrate, in metal-sulfur clusters in proteins (e.g., ferredoxins), and in the regulatory proteins (e.g., thioredoxins) (Figure 2).

## Sulfate transporter and sulfate uptake systems

Most of the sulfate permeases are located in the cytoplasmic membrane. The given Transporter Classification (TC) system consists of nine classes of membrane transporters. TC system is similar to the Enzymes Classification (EC) system; hence, phylogenetic information is also incorporated into this. Most of the known sulfate permeases belong to two transporter classes that include three subclasses: eukaryotic sulfate transporters are categorized in second class, which further divides into two main subclasses (TC 2.A.47. and TC 2.A.53), while prokaryotes possess only one family that belongs to the class third (TC 3.A.1.).^69^^,^^70^

Sulfate transporters are involved in sulfate uptake in eukaryotes, but their role in prokaryotes is not clear.^62^^,^^71^^,^^72^ They transport inorganic anion or perform as anion–anion exchangers. At the same time, some transporters work as sulfate-H^+^ or sulfate-bicarbonate symporters. Many vertebrate SulP homologs have been reported to work as anion–anion antiport. For instance, the mouse homolog, SLC26A6, can transport sulfate, oxalate, formate, bicarbonate, and chloride, exchanging any of these anions for a different one.^73^^,^^74^ They share different affinities with their other substrates. The transport domain of the sulfate promoter comprises 12 to 14 transmembrane helices. For the recruitment of sulfate, positive arginine residues should be present in extracellular loops.^75^^,^^76^ Biochemical studies revealed that sulfate permease functions can also be controlled by phosphorylation after translation.^24^^,^^77^

## Sulfur absorption and transport

**Figure 3.**
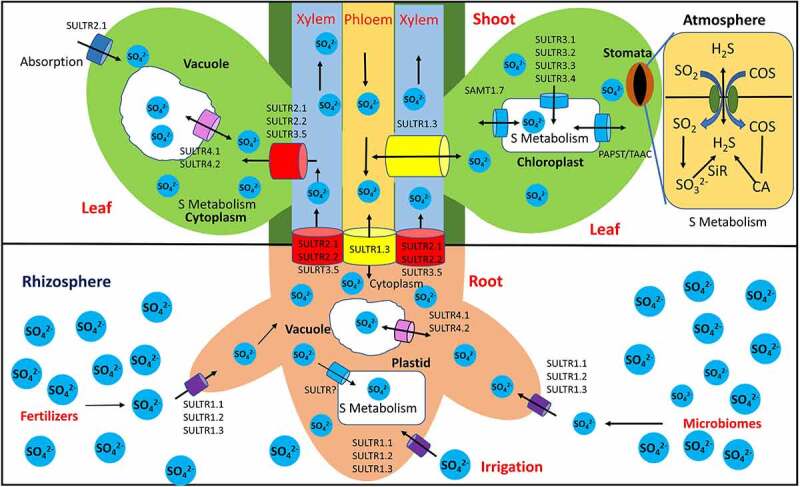
Schematic representation of uptake, transport, assimilation, and storage of sulfate from different sources. Abbreviations; SULTR: H^+^/sulfate cotransporters (indicate by cylindrical shaped diagrams); SAMT: SAM transporter PAPS:3′-Phosphoadenosine 5′-phosphosulfate; PAPST: PAPS transporter, TAAC; Thylakoid ATP/ADP carrier; COS: Carbonyl sulfide; SiR: Sulfite reductase; CA: Carbonic anhydrase. Arrow indicates movement of the sulfate.

Apart from atmospheric sulfur sources like H_2_S, carbonyl sulfide (COS), and SO_2_, mostly sulfur is taken up by dedicated sulfate transporters from the soil in the soluble SO_4_^2−^ ions. This dissolved sulfate is observed by SULTRs, a multigene family H^+^/sulfate co-transporter.^78^ These are high and low-affinity transporters and are distributed in different plants organelles like vacuole, plastid, and chloroplast. High-affinity sulfate transporters (SULTR1;1, SULTR1;2, and SULTR1;3) are most abundant in the epidermis and cortex of the root and facilitate the absorption of sulfate from the soil.^79^ Low-affinity sulfate transporter (SULTR2;1, SULTR2;2, SULTR3;5) are abundant in parenchymatic tissue adjacent to the xylem and phloem. Low-affinity transporters help in the epidermis and the cortex region and work synergistically with high-affinity transporters.^11^^,^^79^ Absorbed SO_4_^2−^ transported to vacuoles with the help of SULTR4;1 and SULTR4;2 as well as distributed to other parts of the plant. These transporters are also helpful in the remobilization of stored sulfate. It has been reported that SULTR1;3, SULTR2;1, SULTR2;2, and SULTR3;5 involved in the transportation of sulfate from root to shoot via xylem^79^ while, SULTR1;3, SULTR2;1, SULTR2;2, and SULTR3;5 involved in the transporting sulfate from root to mesophyll cells of the leaves.^78^ Further, SULTR4;1, and SULTR4;2 helps in transporting sulfate to vacuoles of shoots and leaves and SULTR3;1, SULTR3;2, SULTR3;3, and SULTR3;4 take part in sulfate transport to chloroplasts and further assimilate into other biomolecules.^80^ Atmospheric SO_2_ is absorbed by stomata and converted into SO_3_^2−^ and subsequently takes part in the sulfur reduction pathway in substomatal spaces.^81^ Likewise, atmospheric H_2_S, COS is also taken up by stomata. In leaf mesophyll cells, H_2_S is assimilated by *O*-acetyl-serine (thiol)lyase for the biosynthesis of cysteine. After absorption COS has converted into CO_2_ and H_2_S through carbonic anhydrase (CA).^78^^,^^82^ A schematic representation of uptake, transport, assimilation, and storage of sulfate from different sources at the cellular level has been described in Figure 3.

## Sulfate permeases in fungi

A very little information is available related to fungal sulfate transporters. Some notable fungal sulfate permeases that have been studied belong to *Serendipita indica, Saccharomyces cerevisiae, Neurospora crassa*, and *Penicillium chrysogenum*.^44^^,^^77^^,^^81–85^ Sulfate uptake is a highly regulated step and appears to occur in fungi and plants, via a family of related transporter proteins. It has been shown that enzymes ATP sulfurylase and APS kinase, catalyze the early steps of sulfate assimilation, and of the *Aspergillus* enzyme, cysteine synthase, which produces cysteine from *O*-acetylserine.^86^^,^^88^ Two genes viz., *cys-13* (permease I) and *cys14* (permease II) which encode the *N. crassa* sulfate transporters are of high affinity in nature.^87^^,^^89^ Sulfur sources, like sulfate or methionine, regulate both the genes at the transcriptional level. Under sulfur starvation, both genes have been shown to be highly expressed. Studies in *A. nidulans* have demonstrated that the *sB* gene is located on chromosome VI, whose transcriptional regulation relies on sulfur sources.^90^^,^^91^ Defective *sB* gene strains did not grow on sulfate as the sole sulfur source. However, they grew well on choline sulfate, which is taken up by a different permease. It has been reported the defective strains’ resistant ability to the toxic analogs of sulfate like selenate and chromate, in the condition of non-repressing methionine availability.^92^^,^^93^ In a methionine-supplemented medium, sulfate assimilation, as well as sulfate uptake, is robustly suppressed in *S. cerevisiae, A. nidulans*, and *N. crassa*. However, experiments on mutants compromised with methionine to cysteine conversion indicated that the mutants are regulatory effectors.^90^^,^^94^ This transcriptional regulation is dependent on the sulfur metabolite repression (SMR) system.^93–95 96 97^ Recently, it has been observed that high-affinity sulfate transporter of *S. indica* (SiSulT) uptake sulfate from media and transfer to the host plant. However, the same fungi’ mutant strain fails to uptake and transfer sulfate to the host plant.^44^ Further, it has been shown that *S. indica* SiSulT helped the colonized plant to grow healthy under sulfate-deprived conditions.

## Sulfur signaling and its role in biotic and abiotic stress

Biotic and abiotic stresses adversely affect plant growth and crop productivity. However, nature has evolved several internal mechanisms to cope up with all these stresses. Sulfur plays an important role in different metabolic processes under the normal physiological condition as well as different stress conditions. Sulfur is the constituent of several compounds like amino acids (cysteine and methionine), vitamins (thiamine and biotin), coenzymes, thioredoxin system, glutathione, lipoic acids, and glucosinolates that directly or indirectly take part in ameliorating the adverse effects of different types of biotic and abiotic stresses.^98^ Moreover, sulfur-containing compounds also act as antioxidants that directly modulate the antioxidant defense system in order to save plants from biotic stresses.^98^ Sulfur-containing secondary compounds like sulfolipid and sulfoprotein take part in enzymatic steps related to oxidative stress.^99^ Sulfur-containing amino acids also interact with biomolecules like phytohormones, polyamines, nitric oxide (NO) in order to reduce abiotic stress. Studies suggest that sulfur derivatives accelerate signaling cascades to produce more cellular messengers like NO, Ca^2+^ and abscisic acid which ultimately helps in the initiation of other signaling networks related to stress tolerance.^100,101^^,^^102^ Soil sulfur is taken up by plant roots in a metabolically inert form sulfate (SO_4_^2-^) and it is further processed for its assimilation. The enzyme ATP-sulfurylase (ATP-S) catalyzes the first committed step of sulfur assimilation. It covert SO_4_^2-^ into high-energy compound adenosine-5′-phosphosulfate (APS) and which is further reduced into sulfide (S^2-^) and incorporated into cysteine (Cys).^103^ Further, Cys helps in the synthesis of several sulfur-containing compounds like phytochelatins (PCs), methionine (Met), glutathione (GSH), and homo-GSH (h-GSH). Among them, PCs, GSH, and h-GSH are involved in different abiotic stress tolerance in the plants. Additionally, Met controls the very well-known ethylene signaling for tolerance against the different abiotic stresses through its first metabolite S-adenosylmethionine.^104^ The role, regulation, and underlying mechanisms of ATP-S in plant abiotic and biotic stress tolerance is also emphasized in several studies that indicate its intrinsic regulation by major sulfur-containing compounds.^103^

## H_2_S signaling and its role in sulfur homeostasis

H_2_S is an emerging novel gaseous signaling molecule that takes part in several metabolic processes and signaling in plants. Studies show that H_2_S actively takes part in regulating several physiological processes like seed germination, maturation, senescence as well as overall plant growth and development.^105^ Additionally, it takes part in the plant defense system as well as the acquisition of stress tolerance. Being a gaseous compound, it can easily diffuse in different cellular parts and provide sulfur to the cells and counterbalance the antioxidant pools in cells as well. H_2_S signaling actively takes part in abiotic stresses and increases plant tolerance toward several adverse conditions by activating several associated other mechanisms like oxidative stress signaling, metal transport, Na^+^/K^+^ homeostasis, and antioxidative defense system.^105^ H_2_S also take part in crosstalk in different stress signaling through a complex signaling network that consists of several secondary metabolites and biomolecules such as NO, H_2_O_2_, Ca^2+^, and phytohormones.^106^^,^^107,108^ For instance, the H_2_S signaling initiated by abiotic stress leads to cross induction of signaling against several other stresses like drought, salinity, heavy metal, heat, cold, and flooding stress.^105^ This crosstalk signaling involved induction of several activities such as antioxidant activation, heat shock proteins production, accumulating osmolytes, and maintaining nutrient/ion balance.^109^ Further, during abiotic stress, H_2_S maintains the H_2_S-Cys-cycle which is followed by post-translational modifications of cysteine residues.^105^ At a higher concentration, H_2_S shows cytotoxicity, and at a lower concentration, it shows cell signaling. Therefore, it is very important to maintain H_2_S homeostasis to exert its physiological function as well as crosstalk signaling. Plants have evolved several metabolic mechanisms to maintain the H_2_S homeostasis like other signal molecules.^109^ H_2_S homeostasis is mainly regulated by enzymes like cysteine synthase, sulfite reductase, cyanoalanine synthase, L-cysteine desulfhydrase, and D-cysteine desulfhydrase. L-cysteine desulfhydrase, and D-cysteine desulfhydrase degrades L-/D-cysteine to produce H_2_S. Sulfite reductase convert sulfite to H_2_S. Cyanoalanine synthase catalyzes the H_2_S production from cysteine in the presence of HCN. The formation of cysteine is catalyzed by cysteine synthase in which *O*-acetyl-(thiol)-serinelyase, can incorporate H_2_S into *O*-acetyl-L-serine and its opposite reaction produces H_2_S. So L-cysteine desulfhydrase and D-cysteine desulfhydrase is mainly synthesizing H_2_S in response to different stresses^109^^,^^110^

## Conclusion and future perspective

**Figure 4.**
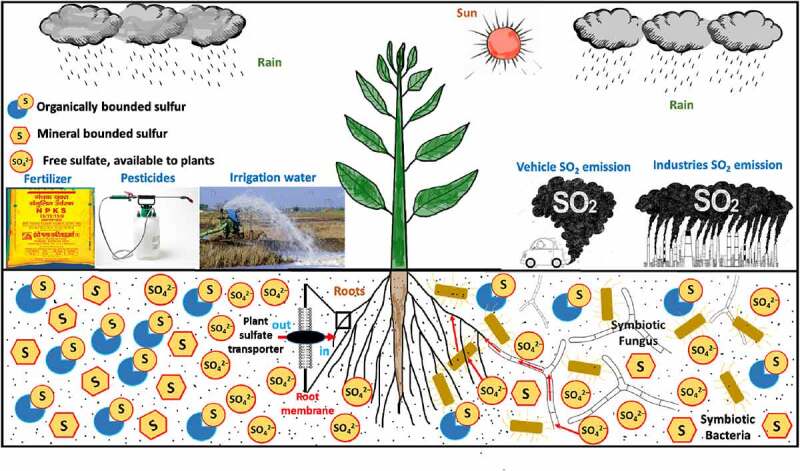
Diagram illustrating the different sources of sulfate. Mainly free sulfate ions are a bioavailable form of sulfur. Plant uptake these free sulfate ions via their transporters. Symbiotic fungus and bacteria also help plants in the acquisition of sulfate from the soil rhizosphere.

Sulfur nutrition is essential for the growth and development of plants. Sulfur deficiency leads to retarded growth and yield. Sulfate permeases of plants and plant-associated organisms (fungi and bacteria) play a crucial role in sulfur uptake from soil. Plants are able to take up sulfate from the soil over a wide range of concentrations through the use of high-affinity and low-affinity transporters.^10^^,^^62^ These sulfate transporters belong to the major facilitator superfamily (MFS) group of membrane transport proteins. As mentioned in soils with low sulfur availability, a symbiotic association between plants and an AMF assists with the sulfur acquisition from the soil: plants obtain nutrients from their fungal partner, which in return receives sugars from the plant.^40^^,^^111^ In this association, fungal and plant membrane transporters participate in nutrient transfer to the host plant. However, due to the lack of a stable transformation system in the case of AMF, the sulfate transporter system of AMF could not be manipulated to improve sulfur uptake in colonized plants. On the other hand, the beneficial endophyte *S. indica* can be cultivated axenically under laboratory conditions and has a well-established transformation system therefore functions of various genes have been studied.^45^^,^^112^^,^^113^ Colonization by *S. indica* improves a plant’s ability to acquire phosphorus, magnesium, iron, and sulfate from a nutrient-deprived soil rhizosphere,^44^^,^^45^^,^^114^^,^^115 116 117 118 119^ due to the presence of dedicated nutrient transporters providing benefits to the host plant such as improved growth and increased resistance to biotic and abiotic stresses.^113^^,^^112–^^115^ Therefore, *S. indica* has been termed a plant probiotic.^120^ The versatile potential of *S. indica* makes it a promising agent in agricultural applications. Understanding the mechanism that *S. indica* utilizes to improve plant growth opens exciting avenues to further improve the fungal talents. In our opinion, despite all these novel approaches to improve sulfur enrichment in plants, detailed studies on the sulfate permeases and high-affinity sulfate transporters from the plant side as well as from associated partners are highly required to encourage sustainable agriculture and to reduce the load of chemical fertilizers. The crosstalk between plant and fungal/bacterial partners at the molecular level is less known. Hence, future studies on inter-organismic nutrient transfer can open new vistas to improve the nutrient exchange and hence plant growth and development. Sulfur and sulfur-containing compounds have been playing important role in the growth and development of plants. They play role in catalyzing several metabolic processes, as well as a cross mediator in different biotic and abiotic stress tolerance. Sulfur deficiency in the soil became the key factor limiting crop growth and yield. So far, as compared to other nutrients, studies on sulfur absorption, metabolism, regulation, and its mechanistic understanding is not enough and remain obscure. Thus, in the future, to improve the sulfur utilization efficiency in plants, more effort is required to know the regulatory mechanisms of plant’s response toward sulfur deficiency in soil and different stresses. The summary of this study is described in Figure 4.

## References

[1] Li Q, Gao Y, Yang A. Sulfur homeostasis in plants. Int J Mol Sci. 2020;23:8926. doi:10.3390/ijms21238926. PMC7727837 33255536

[2] Nakai Y, Maruyama-Nakashita A. Biosynthesis of sulfur-containing small biomolecules in plants. Int J Mol Sci. 2020;21(10):3470. doi:10.3390/ijms21103470. 32423011 PMC7278922

[3] Scherer HW, Pacyna S, Spoth KR, Schulz M. Low levels of ferredoxin, ATP and leghemoglobin contribute to limited N_2_ fixation of peas (*Pisum sativum L*.) and alfalfa (*Medicago sativa L*.) under S deficiency conditions. Biol and Fertility Soils. 2008;44(7):909–11. doi:10.1007/s00374-008-0273-7.

[4] Aarabi F, Naake T, Fernie AR, Hoefgen R. Coordinating sulfur pools under sulfate deprivation. Trends Plant Sci. 2020;25:1227–1239. doi:10.1016/j.tplants.2020.07.007. 32800669

[5] Leustek T, Saito K. Sulfate transport and assimilation in plants. Plant Physiol. 1999;120(3):637–644. doi:10.1104/pp.120.3.637. 10398698 PMC1539218

[6] Gohain BP, Rose TJ, Liu L, Barkla BJ, Raymond CA, King GJ. Remobilization and fate of sulphur in mustard. Ann Bot. 2019 August 16;124(3):471–480. doi:10.1093/aob/mcz101. 31181139 PMC6798836

[7] Jogawat A, Yadav B, Narayan OP. Metal transporters in organelles and their roles in heavy metal transportation and sequestration mechanisms in plants. Physiol Plant. 2021b. doi:10.1111/ppl.13370. 33586164

[8] Yadav B, Jogawat A, Lal SK, Lakra N, Mehta S, Shabek N, and Narayan OP. Plant mineral transport systems and the potential for crop improvement. Planta. 2021a;253:45. doi:10.1007/s00425-020-03551-7. 33483879

[9] Bashir K, Ishimaru Y, Shimo H, Nagasaka S, Fujimoto M, Takanashi H, Tsutsumi N, An G, Nakanishi H, Nishizawa NK. The rice mitochondrial iron transporter is essential for plant growth. Nat Commun. 2011;2:322. doi:10.1038/ncomms1326. 21610725 PMC3113228

[10] Leustek T, Martin MN, Bick JA, Davies JP. Pathways and regulation of sulfur metabolism revealed through molecular and genetic studies. Ann Review Plant Biol. 2000;51(1):141–165. doi:10.1146/annurev.arplant.51.1.141. 15012189

[11] Takahashi H, Kopriva S, Giordano M, Saito K, Hell R. Sulfur assimilation in photosynthetic organisms: molecular functions and regulations of transporters and assimilatory enzymes. Annual Rev Plant Biol. 2011;62:157–184. doi:10.1146/annurev-arplant-042110-103921. 21370978

[12] Tabatabai MA, Bremner JM. Distribution of total and available sulfur in selected soils and soil profiles. Agron J. 1972;64(1):40–44. doi:10.2134/agronj1972.00021962006400010013x.

[13] Blum SC, Lehmann J, Solomon D, Caires EF, Alleoni LR. Sulfur forms in organic substrates affecting S mineralization in soil. Geoderma. 2013;200:156–164. doi:10.1016/j.geoderma.2013.02.003.

[14] Uroz S, Calvaruso C, Turpault MP, Frey-Klett P. Mineral weathering by bacteria: ecology, actors and mechanisms. Trends Microbiol. 2009;17(8):378–387. doi:10.1016/j.tim.2009.05.004. 19660952

[15] Feinberg A, Stenke A, Peter T, Hinckley EL, Driscoll CT, Winkel LH. Reductions in the deposition of sulfur and selenium to agricultural soils pose risk of future nutrient deficiencies. Commun Earth Environ. 2021;2(1):1–8. doi:10.1038/s43247-021-00172-0.

[16] Craig K. A review of the chemistry, pesticide use, and environmental fate of sulfur dioxide, as used in California. Rev Environ Contam Toxicol. 2018;246(246):33–64. doi:10.1007/398_2018_11. 29526018

[17] Griffith CM, Woodrow JE, Seiber JN. Environmental behavior and analysis of agricultural sulfur. Pest Manag Sci. 2015;71(11):1486–1496. doi:10.1002/ps.4067. 26108794

[18] Etienne P, Sorin E, Maillard A, Gallardo K, Arkoun M, Guerrand J, Cruz F, Yvin JC, Ourry A. Assessment of sulfur deficiency under field conditions by single measurements of sulfur, chloride and phosphorus in mature leaves. Plants. 2018;7(2):37. doi:10.3390/plants7020037. 29710786 PMC6027431

[19] McGrath SP, Zhao FJ, Withers PJA. Development of sulphur deficiency in crops and its treatment. Proceedings-Fertiliser Society (United Kingdom).1996.

[20] Hawkesford MJ, Kopriva S, De Kok LJ. Nutrient use efficiency in plants. Springer International Pu; 2016. doi:10.1007/978-3-319-10635-9.

[21] Yu Z, She M, Zheng T, Diepeveen D, Islam S, Zhao Y, Zhang Y, Tang G, Zhang Y, Zhang J, et al. Impact and mechanism of sulphur-deficiency on modern wheat farming nitrogen-related sustainability and gliadin content. Comm Bio. 2021;4(1):1–6. doi:10.1038/s42003-021-02458-7. PMC8346565 34362999

[22] Gilbert SM, Clarkson DT, Cambridge M, Lambers H, Hawkesford MJ. SO_4_^2-^deprivation has an early effect on the content of ribulose-1, 5-bisphosphate carboxylase/oxygenase and photosynthesis in young leaves of wheat. Plant Physiol. 1997;115(3):1231–1239. doi:10.1104/pp.115.3.1231. 12223869 PMC158588

[23] Jobe TO, Zenzen I, Rahimzadeh Karvansara P, and Kopriva S. Integration of sulfate assimilation with carbon and nitrogen metabolism in transition from C3 to C4 photosynthesis. J Exp Bot. 2019;70(16):4211–4221. doi:10.1093/jxb/erz250. 31124557 PMC6698703

[24] Mitchell SC. Nutrition and sulfur. In: Advances in food and nutrition research. Vol. 96. Academic Press; 2021 Jan 1. p. 123–174. doi:10.1016/bs.afnr.2021.02.014. 34112351

[25] Houhou M, Joutei KA, Louhalia S. Biomass production, chlorophyll content and morphorogical parameters are affected by sulfur deficiency in *Eruca sativa* L. Int J Ecol Environ Sci. 2018;44:67–75.

[26] Filipek-Mazur B, Tabak M, Gorczyca O, Lisowska A. Effect of sulfur-containing fertilizers on the quantity and quality of spring oilseed rape and winter wheat yield. J Elementol. 2019;24(4). doi:10.5601/jelem.2019.24.1.1809.

[27] Karmoker JL, Clarkson DT, Saker LR, Rooney JM, Purves JV. Sulphate deprivation depresses the transport of nitrogen to the xylem and the hydraulic conductivity of barley (*Hordeum vulgare L*.) roots. Planta. 1991;185(2):269–278. doi:10.1007/bf00194070. 24186351

[28] Carciochi WD, Divito GA, Fernández LA, Echeverría HE. Sulfur affects root growth and improves nitrogen recovery and internal efficiency in wheat. J Plant Nutrition. 2017;40(9):1231–1242. doi:10.1080/01904167.2016.1187740.

[29] Haneklaus S, Bloem E, Schnug E, De Kok LJ, Stulen I. Sulfur. In: Barker AV, Pilbeam DJ, editors. Handbook of plant nutrition. CRC press; 2007. p. 183–238.

[30] Camberato J, Casteel S. Keep an eye open for sulfur deficiency in wheat. Fertility update, dept of agronomy. Purdue university; 2010.

[31] Gao Y, Li X, Tian QY, Wang BL, Zhang WH. Sulfur deficiency had different effects on *Medicago truncatula* ecotypes A17 and R108 in terms of growth, root morphology and nutrient contents. J Plant Nutr. 2016;39:301–314. doi:10.1080/01904167.2014.976344.

[32] Klimont Z, Smith SJ, Cofala J. The last decade of global anthropogenic sulfur dioxide: 2000–2011 emissions. Environ Res Lett. 2013;8(1):014003. doi:10.1088/1748-9326/8/1/014003.

[33] Santana MM, Gonzalez JM, Clara MI. Inferring pathways leading to organic-sulfur mineralization in the Bacillales. Crit Rev Microbiol. 2016;42(1):31–45. doi:10.3109/1040841X.2013.877869. 24506486

[34] Zublena JP, Baird JV, Lilly JP. Soil facts-nutrient content of fertilizer and organic materials. North Carolina Cooperative Extension Service. 1991.

[35] Sawyer JE, Lang B, Barker DW. Sulfur fertilization response in Iowa corn and soybean production. 2012;51:39‐48.

[36] Messick DL, Fan MX, De Brey C. Global sulfur requirement and sulfur fertilizers. FAL—Agric Res. 2005;283:97–104.

[37] Camberato J, Casteel S. Sulfur deficiency. Purdue Univ Dep of Agronomy, Soil Fertility Update. 2017.

[38] Kurmanbayeva M, Sekerova T, Tileubayeva Z, Kaiyrbekov T, Kusmangazinov A, Shapalov S, Madenova A, Burkitbayev M, Bachilova N. Influence of new sulfur-containing fertilizers on performance of wheat yield. Saudi J Bio Sci. 2021;(8):4644–4655. doi:10.1016/j.sjbs.2021.04.073. 34354451 PMC8324966

[39] Sun Z, Song J, Xin XA, Xie X, Zhao B. Arbuscular mycorrhizal fungal 14-3-3 proteins are involved in arbuscule formation and responses to abiotic stresses during AM symbiosis. Front Microbiol. 2018;9:91. doi:10.3389/fmicb.2018.00091. 29556216 PMC5844941

[40] Begum N, Qin C, Ahanger MA, Raza S, Khan MI, Ashraf M, Ahmed N, Zhang L. Role of arbuscular mycorrhizal fungi in plant growth regulation: implications in abiotic stress tolerance. Front Plant Sci. 2019;10:1068. doi:10.3389/fpls.2019.01068. 31608075 PMC6761482

[41] Wang B, Qiu YL. Phylogenetic distribution and evolution of mycorrhizas in land plants. Mycorrhiza. 2006;16(5):299–363. doi:10.1007/s00572-005-0033-6. 16845554

[42] Morton JB, Benny GL. Revised classification of arbuscular mycorrhizal fungi (Zygomycetes): a new order, *Glomales*, two new suborders, *Glomineae* and *Gigasporineae*, and two new families, *Acaulosporaceae* and *Gigasporaceae*, with an emendation of *Glomaceae*. Mycotaxon. 1990;37:471–491. doi:10.1017/S0953756200002860.

[43] Nagahashi G, Douds DD. Partial separation of root exudate components and their effects upon the growth of germinated spores of AM fungi. Mycol Res. 2000;104(12):1453–1464. doi:10.1017/S0953756200002860.

[44] Narayan OP, Verma N, Jogawat A, Dua M, Johri AK. Sulfur transfer from the endophytic fungus *Serendipita indica* improves maize growth and requires the sulfate transporter SiSulT. Plant Cell. 2021;33:1268–1285. doi:10.1093/plcell/koab006. 33793849

[45] Verma N, Narayan OP, Prasad D, Jogawat A, Panwar SL, Dua M, Johri AK. Functional characterization of a high affinity iron transporter (*PiFTR*) from the endophytic fungus *Piriformospora indica* and its role in plant growth and development. Environ Microbiol. 2021. doi:10.1111/1462-2920.15659. 34227231

[46] Buchner P, Takahashi H, Hawkesford MJ. Plant sulphate transporters: co-ordination of uptake, intracellular and long-distance transport. J Exp Bot. 2004;55(404):1765–1773. doi:10.1093/jxb/erh206. 15258169

[47] Kertesz MA, Fellows E, Schmalenberger A. Rhizobacteria and plant sulfur supply. Adv Appl Microbiol. 2007;62:235–268. doi:10.1016/S0065-2164(07)62008-5. 17869607

[48] Giovannetti M, Tolosano M, Volpe V, Kopriva S, Bonfante P. Identification and functional characterization of a sulfate transporter induced by both sulfur starvation and mycorrhiza formation in *Lotus japonicus*. New Phytol. 2014;204(3):609–619. doi:10.1111/nph.12949. 25132489

[49] Gryndler M, Hršelová H, Stříteská D. Effect of soil bacteria on hyphal growth of the arbuscular mycorrhizal fungus *Glomus claroideum*. Folia Microbiol. 2000;45(6):545–551. doi:10.1007/BF02818724. 11501421

[50] Siciliano SD, Palmer AS, Winsley T, Lamb E, Bissett A, Brown MV, van Dorst J, Ji M, Ferrari BC, Grogan P, Chu H, Snape I. Soil fertility is associated with fungal and bacterial richness, whereas pH is associated with community composition in polar soil microbial communities. Soil Biol Biochem. 2014;78:10–20. doi:10.1016/j.soilbio.2014.07.005.

[51] Gahan J, Schmalenberger A. Bacterial and fungal communities in the mycorrhizospheres of *Agrostis, Lolium* and *Plantago* respond to inoculation with arbuscular mycorrhizal fungi. In: Diskin MG, editor. Agricultural research forum. Vol. 2013. Tullamore: Teagasc; 2013. p. 4.

[52] Gahan J, Schmalenberger A. Arbuscular mycorrhizal hyphae in grassland select for a diverse and abundant hyphospheric bacterial community involved in sulfonate desulfurization. Applied Soil Ecology. 2015;89:113–121. doi:10.1016/j.apsoil.2014.12.008.

[53] Vilarino A, Frey B, Shüepp H. MES [2-(N-morpholine)-ethane sulphonic acid] buffer promotes the growth of external hyphae of the arbuscular mycorrhizal fungus *Glomus intraradices* in an alkaline sand. Biology and Fertility of Soils. 1997;25(1):79–81. doi:10.1007/s003740050284.

[54] Abdel-Rahman SSA, El-Naggar A-RI. Promotion of rooting and growth of some types of bougainvilleas cutting by plant growth promoting rhizobacteria (pgpr) and arbuscular mycorrhizal fungi (amf) in combination with Indole-3-Butyric Acid (IBA) 2014. Ijsr. 3:97–108.

[55] Andrade G, Mihara KL, Linderman RG, Bethlenfalvay GJ. Soil aggregation status and rhizobacteria in the mycorrhizosphere. Plant Soil. 1998;202(1):89–96. doi:10.1023/A:1004301423150.

[56] Linderman RG. Mycorrhizal interactions with the rhizosphere microflora: the mycorrhizosphere effect. Phytopathology. 1991;78(3):366–371. doi:10.1007/978-94-011-3336-4_73.

[57] Bonkowski M. Protozoa and plant growth: the microbial loop in soil revisited. New Phytol. 2004;162(3):617–631. doi:10.1111/j.1469-8137.2004.01066.x. 33873756

[58] Irshad U, Villenave C, Brauman A, Plassard C. Grazing by nematodes on rhizosphere bacteria enhances nitrate and phosphorus availability to *Pinus pinaster* seedlings. Soil Biol Biochem. 2011;43(10):2121–2126. doi:10.1016/j.soilbio.2011.06.015.

[59] Mitra D, Uniyal N, Panneerselvam P, Senapati A, Ganeshamurthy AN. Role of mycorrhiza and its associated bacteria on plant growth promotion and nutrient management in sustainable agriculture. Ijlsas. 2019;1:1.

[60] Agrawal M. Plant responses to atmospheric sulphur. In: Y.p A, Ahmad A, editors. Sulphur in plants. Dordrecht: Springer; 2003. p. 279–293. doi:10.1007/978-94-017-0289-8_15.

[61] De Kok LJ, Tausz M. The role of glutathione in plant reaction and adaptation to air pollutants. In: Significance of glutathione to plant adaptation to the environment. Netherlands: Springer; 2001. p. 185–205. doi:10.1007/0-306-47644-4_8.

[62] Takahashi H. Sulfate transport systems in plants: functional diversity and molecular mechanisms underlying regulatory coordination. J Exp Bot. 2019;70(16):4075–4087. doi:10.1093/jxb/erz132. 30907420

[63] De Kok LJ, Stuiver CEE, Westerman S, Stulen I. Elevated levels of hydrogen sulfide in the plant environment: nutrient or toxin. In: Air pollution and plant biotech. Japan: Springer; 2002. p. 201–219. doi :10.1007/978-4-431-68388-9_10.

[64] Heinz E. Recent investigations on the biosynthesis of the plant sulfolipid. sulfur nutrition and assimilation in higher plants. Academic Publishing Bv. 1993;163–178.

[65] Cram WJ. Uptake and transport of sulfate. Sulfur nutrition and sulfur assimilation in higher plants: fundamental, environmental and agricultural aspects. SPB Academic Publishing bv; 1990. p. 3–11.

[66] Kopriva S, Koprivova A. Sulphate assimilation: a pathway which likes to Surprise. In: Sulphur in plants. Netherlands: Springer; 2003. p. 87–112. doi:10.1007/978-94-017-0289-8_5.

[67] Sbodio JI, Snyder SH, Paul BD. Regulators of the transsulfuration pathway. Br J Pharmacol. 2019;176(4):583–593. doi:10.1111/bph.14446. 30007014 PMC6346075

[68] Kopriva S, Malagoli M, Takahashi H. Sulfur nutrition: impacts on plant development, metabolism, and stress responses. J Exp Bot. 2019;70(16):4069–4073. doi:10.1093/jxb/erz319. 31423538

[69] Marietou A, Røy H, Jørgensen BB, Kjeldsen KU. Sulfate transporters in dissimilatory sulfate reducing microorganisms: a comparative genomics analysis. Front Microbiol. 2018;9:309. doi:10.3389/fmicb.2018.00309. 29551997 PMC5840216

[70] Milton H, Reddy VJ, Tamang D, Västermark A. The transporter classification database. Nucleic Acids Res. 2014;4(2):251–258. doi:10.1093/nar/gkt1097. PMC3964967 24225317

[71] Sandal NN, Marcker KA. Similarities between a soybean nodulin, *Neurospora crassa* sulphate permease II and a putative tumor suppressor. Trends Biochem Sci. 1994;19:19. doi:10.1016/0968-0004(94)90168-6. 8140616

[72] Smith FW, Hawkesford MJ, Prosser IM, Clarkson D. Isolation of a cDNA from *Saccharomyces cerevisiae* that encodes a high affinity sulphate transporter at the plasma membrane. Mol General Gen. 1995b;247(6):709–715. doi:10.1007/BF00290402. 7616962

[73] Alper SL, Sharma AK. The SLC26 gene family of anion transporters and channels. Mol Aspects Med. 2013;34(2–3):494–515. doi:10.1016/j.mam.2012.07.009. 23506885 PMC3602804

[74] Jiang Z, Grichtchenko II, Boron WF, and Aronson PS. Specificity of anion exchange mediated by mouse Slc26a6. J Biol Chem. 2002;277(37):33963–33967. doi:10.1074/jbc.M202660200. 12119287

[75] Smith FW, Ealing PM, Hawkesford MJ, and Clarkson DT. Plant members of a family of sulfate transporters reveal functional subtypes. Proc Natl Acad Sci USA. 1995a;92(20):9373–9377. doi:10.1073/pnas.92.20.9373. 7568135 PMC40987

[76] Wang L, Chen K, Zhou M. Structure and function of an *Arabidopsis thaliana* sulfate transporter. Nat Comm. 2021;12(1):1–8. doi:10.1038/s41467-021-24778-2. PMC8298490 34294705

[77] Breton A, Surdin-Kerjan Y. Sulfate uptake in *Saccharomyces cerevisiae*: biochemical and genetic study. J Bacteriol. 1977;132(1):224–232. doi:10.1128/JB.132.1.224-232.1977. 199574 PMC221848

[78] Fuentes-Lara LO, Medrano-Macías J, Pérez-Labrada F, Rivas-Martínez EN, García-Enciso EL, González-Morales S, Juárez-Maldonado A, Rincón-Sánchez F, Benavides-Mendoza A. From elemental sulfur to hydrogen sulfide in agricultural soils and plants. Molecules. 2019;24(12):2282. doi:10.3390/molecules24122282. 31248198 PMC6630323

[79] Maruyama-Nakashita A. Metabolic changes sustain the plant life in low-sulfur environments. Curr Opin Plant Biol. 2017;39:144–151. doi:10.1016/j.pbi.2017.06.015. 28759781

[80] Cao MJ, Wang Z, Wirtz M, Hell R, Oliver DJ, Xiang CB. SULTR 3; 1 is a chloroplast‐localized sulfate transporter in *Arabidopsis thaliana*. Plant J. 2013;73(4):607–616. doi:10.1111/tpj.12059. 23095126

[81] Aghajanzadeh T, Hawkesford MJ, De Kok LJ. Atmospheric H_2_S and SO_2_ as sulfur sources for *Brassica juncea* and *Brassica rapa*: regulation of sulfur uptake and assimilation. Environ Exp Bot. 2016;124:1. doi:10.1016/j.envexpbot.2015.12.001.

[82] Stimler K, Montzka SA, Berry JA, Rudich Y, Yakir D. Relationships between carbonyl sulfide (COS) and CO_2_ during leaf gas exchange. New Phytol. 2010;186(4):869–878. doi:10.1111/j.1469-8137.2010.03218.x. 20298480

[83] Cherest H, Davidian JC, Thomas D, Benes V, Ansorge W, Surdin-Kerjan Y. Molecular characterization of two high affinity sulfate transporters in *Saccharomyces cerevisiae*. Genetics. 1997;145(3):627–635. doi:10.1093/genetics/145.3.627. 9055073 PMC1207848

[84] Ketter JS, Marzluf GA. Molecular cloning and analysis of the regulation of *cys-14+*, a structural gene of the sulfur regulatory circuit of *Neurospora crassa*. Mol Cell Biol. 1988;8(4):1504–1508. doi:10.1128/mcb.8.4.1504. 2898097 PMC363309

[85] Marzluf GA. Molecular genetics of sulfur assimilation in filamentous fungi and yeast. Ann Rev Microbiol. 1997;51(1):73–96. doi:10.1146/annurev.micro.51.1.73. 9343344

[86] Van De Kamp M, Pizzinini E, Vos A, van der Lende TR, Schuurs TA, Newbert RW, Driessen AJ, Konings WN, Driessen AJM. Sulfate Transport in Penicillium chrysogenum: cloning and characterization of the sutA and sutB Genes. J Bacterial. 1999;181(23):7228–7234. doi:10.1128/JB.181.23.7228-7234.1999. PMC103684 10572125

[87] Van De Kamp M, Schuurs TA, Vos A, van der Lende TR, Konings WN, Driessen AJ. Sulfur regulation of the sulfate transporter genes *sutA* and *sutB* in *Penicillium chrysogenum*. App Environ Microbiol. 2000;66(10):4536–4538. doi:10.1128/aem.66.10.4536-4538.2000. PMC92338 11010912

[88] Linder T. Assimilation of alternative sulfur sources in fungi. World J Microbiol Biotechnol. 2018;34(4):1–7. doi:10.1007/s11274-018-2435-6. PMC5857272 29550883

[89] Huberman LB, Wu VW, Lee J, Daum C, O’Malley RC, Glass NL. Aspects of the Neurospora crassa sulfur starvation response are revealed by transcriptional profiling and DNA affinity purification sequencing. Msphere. 2021;6(5):e00564–21. doi:10.1128/mSphere.00564-21. 34523983 PMC8550094

[90] Piłsyk S, Mieczkowski A, Golan MP, Wawrzyniak A, Kruszewska JS. Internalization of the *Aspergillus nidulans* AstA Transporter into Mitochondria Depends on Growth Conditions, and Affects ATP Levels and Sulfite Oxidase Activity. Int J Mol Sci. 2020;21(20):7727. doi:10.3390/ijms21207727. 33086570 PMC7589619

[91] Piłsyk S, Natorff R, Sieńko M, Paszewski A. Sulfate transport in *Aspergillus nidulans*: a novel gene encoding alternative sulfate transporter. Fungal Gen Biol. 2007;44(8):715–725. doi:10.1016/j.fgb.2006.11.007. 17223367

[92] Arst HN. Genetic analysis of the first steps of sulphate metabolism in *Aspergillus nidulans*. Nature. 1968;219(5151):268–270. doi:10.1038/219268a0. 5671427

[93] Viti C, Marchi E, Decorosi F, Giovannetti L. Molecular mechanisms of Cr (VI) resistance in bacteria and fungi. FEMS Microbiol Rev. 2014;38(4):633–659. doi:10.1111/1574-6976.12051. 24188101

[94] Hansen J, Francke Johannesen P. Cysteine is essential for transcriptional regulation of the sulfur assimilation genes in *Saccharomyces cerevisiae*. Mol General Genet. 2000;263(3):535–542. doi:10.1007/s004380051199. 10821189

[95] Chen Y, Zhang Z, Li B, Tian S. PeMetR-mediated sulfur assimilation is essential for virulence and patulin biosynthesis in *Penicillium expansum*. Environ Microbiol. 2021;23(9):5555–5568. doi:10.1111/1462-2920.15704. 34347341

[96] Natorff R, Sieńko M, Brzywczy J, Paszewski A. The *Aspergillus nidulans metR* gene encodes a bZIP protein which activates transcription of sulphur metabolism genes. Mol Microbiol. 2003;49(4):1081–1094. doi:10.1046/j.1365-2958.2003.03617.x. 12890030

[97] Paszewski A, Natorff R, Piotrowska M, Brzywczy J, Sienko M, Grynberg M, Turner G. Regulation of sulfur amino acid biosynthesis in *Aspergillus nidulans*: physiological and genetical aspects. Sulfur nutrition and sulfur assimilation in higher plants. Bern: Paul Haupt Publishers; 2000;93–105. doi:10.1007/978-3-662-06064-3_18.

[98] Samanta S, Singh A, Roychoudhury A. Involvement of sulfur in the regulation of abiotic stress tolerance in plants. Protective chemical agents in the amelioration of plant abiotic stress. Biochemical and Molecular Perspectives. 2020;22:437–466. doi:10.1002/9781119552154.ch22.

[99] Adak MK, Saha I, Dolui D, Debnath SC. Sulfur in soil: abiotic stress signaling, transmission and induced physiological responses in plants. In: Soil science: fundamentals to recent advances. Singapore: Springer; 2021. p. 469–492. doi:10.1007/978-981-16-0917-6_24.

[100] Hasanuzzaman M, Bhuyan MH, Mahmud JA, Nahar K, Mohsin SM, Parvin K, Fujita M. Interaction of sulfur with phytohormones and signaling molecules in conferring abiotic stress tolerance to plants. Plant Signal Behav. 2018;13(5):e1477905. doi:10.1080/15592324.2018.1477905. 29939817 PMC6103289

[101] Yadav B, Dubey R, Gnanasekaran P, Narayan OP. OMICS approaches towards understanding plant's responses to counterattack heavy metal stress: An insight into molecular mechanisms of plant defense. Plant Gene. 2021b;28:100333. doi:10.1016/j.plgene.2021.100333.

[102] Yadav B, Jogawat A, Gnanasekaran P, Kumari P, Lakra N, Lal SK, Pawar J, Narayan OP. An overview of recent advancement in phytohormones-mediated stress management and drought tolerance in crop plants. Plant Gene. 2021c;25:100264. doi:10.1016/j.plgene.2020.100264.

[103] Anjum NA, Gill R, Kaushik M, Hasanuzzaman M, Pereira E, Ahmad I, Tuteja N, Gill SS. ATP-sulfurylase, sulfur-compounds, and plant stress tolerance. Front Plant Sci. 2015;6:210. doi:10.3389/fpls.2015.00210. 25904923 PMC4387935

[104] Jogawat A, Yadav B, Lakra N, Singh AK, and Narayan OP. Crosstalk between phytohormones and secondary metabolites in the drought stress tolerance of crop plants: a review. Physiol Plant. 2021a;172(2):1106–1132. doi:10.1111/ppl.13328. 33421146

[105] Khanna K, Sharma N, Kour S, Ali M, Ohri P, Bhardwaj R. Hydrogen Sulfide: a robust combatant against abiotic stresses in plants. Hydrogen. 2021;2(3):319–342. doi:10.3390/hydrogen2030017.

[106] Siddiqui MH, Alamri S, Mukherjee S, Al-Amri AA, Alsubaie QD, Al-Munqedhi BM, Ali HM, Kalaji HM, Fahad S, Rajput VD, and Narayan, OP. Molybdenum and hydrogen sulfide synergistically mitigate arsenic toxicity by modulating defense system, nitrogen and cysteine assimilation in faba bean (*Vicia faba* L.) seedlings. Environ Pollut. 2021;290:117953. doi:10.1016/j.envpol.2021.117953. 34438168

[107] Wang L, Wan R, Shi Y, Xue S. Hydrogen sulfide activates S-type anion channel via OST1 and Ca^2+^ modules. Mol Plant. 2016;9(3):489–491. doi:10.1016/j.molp.2015.11.010. 26678664

[108] Yadav B, Jogawat A, Rahman MS, and Narayan OP. Secondary metabolites in the drought stress tolerance of crop plants: A review. Gene Rep. 2021d;23:101040. doi:10.1016/j.genrep.2021.101040. 33421146

[109] Li ZG, Min X, Zhou ZH. Hydrogen sulfide: a signal molecule in plant cross-adaptation. Front Plant Sci. 2016;7:1621. doi:10.3389/fpls.2016.01621. 27833636 PMC5080339

[110] Hancock JT, Whiteman M. Hydrogen sulfide and cell signaling: team player or referee?. Plant Physiol Biochem. 2014;78:37–42. doi:10.1016/j.plaphy.2014.02.012. 24607577

[111] Parniske M. Arbuscular mycorrhiza: the mother of plant root endosymbioses. Nat Rev Microbiol. 2008;6:763. doi:10.1038/nrmicro1987. 18794914

[112] Akum FN, Steinbrenner J, Biedenkopf D, Imani J, Kogel KH. The *Piriformospora indica* effector PIIN_08944 promotes the mutualistic Sebacinalean symbiosis. Front Plant Sci. 2015;6:906. doi:10.3389/fpls.2015.00906. 26579156 PMC4620400

[113] Jogawat A, Vadassery J, Verma N, Oelmüller R, Dua M, Nevo E, Johri AK. PiHOG1, a stress regulator MAP kinase from the root endophyte fungus *Piriformospora indica*, confers salinity stress tolerance in rice plants. Sci Rep. 2016;6:36765. doi:10.1038/srep36765. 27849025 PMC5111105

[114] Kumar M, Yadav V, Kumar H, Sharma R, Singh A, Tuteja N, Johri AK. *Piriformospora indica* enhances plant growth by transferring phosphate. Plant Signal Behavior. 2011;6(5):723–725. doi:10.4161/psb.6.5.15106. PMC3172848 21502815

[115] Prasad D, Verma N, Bakshi M, Narayan OP, Singh AK, Dua M, Johri AK. Functional characterization of a magnesium transporter of root endophytic fungus *Piriformospora indica*. Front Microbiol. 2019;9:3231. doi:10.3389/fmicb.2018.03231. 30687249 PMC6333687

[116] Johri AK, Oelmüller R, Dua M, Yadav V, Kumar M, Tuteja N, Varma A, Bonfante P, Persson BL, Stroud RM. Fungal association and utilization of phosphate by plants: success, limitations, and future prospects. Front Microbiol. 2015;6:984. doi:10.3389/fmicb.2015.00984. 26528243 PMC4608361

[117] Kumar M, Yadav V, Tuteja N, Johri AK. Antioxidant enzyme activities in maize plants colonized with *Piriformospora indica*. Microbiol. 2009;155:780–790. doi:10.1099/mic.0.019869-0. 19246749

[118] Narayan OP, Verma N, Singh AK, Oelmüller R, Kumar M, Prasad D, Kapoor R, Dua M, Johri AK. Antioxidant enzymes in chickpea colonized by *Piriformospora indica* participate in defense against the pathogen *Botrytis cinerea*. Sci Rep. 2017;7:13553. doi:10.1038/s41598-017-12944-w. 29051515 PMC5648792

[119] Waller F, Achatz B, Baltruschat H, Fodor J, Becker K, Fischer M, Heier T, Hückelhoven R, Neumann C, von Wettstein D, Franken, P, Kogel, KH. The endophytic fungus *Piriformospora indica* reprograms barley to salt-stress tolerance, disease resistance, and higher yield. Proc Natl Acad Sci USA. 2005;102:13386–13391. doi:10.1073/pnas.0504423102. 16174735 PMC1224632

[120] Aschheim K, Cervoni N, DeFrancesco L, Hare P, Taroncher-Oldenburg G. Plant probiotic (News and Views). Nat Biotech. 2005;23:10.1038.

